# Relationship among positive self-esteem, physical literacy, and physical activity in college students: a study of a mediation model

**DOI:** 10.3389/fpsyg.2023.1097335

**Published:** 2023-05-17

**Authors:** Xi She, Tian-Yu Gao, Rui-Si Ma, Di Tang, Hua Zhong, He-Ling Dong

**Affiliations:** ^1^School of Physical Education, South China University of Technology, Guangzhou, China; ^2^School of Physical Education, Jinan University, Guangzhou, China; ^3^Department of Sports Science and Physical Education, Faculty of Education, The Chinese University of Hong Kong, Shatin, Hong Kong SAR, China

**Keywords:** positive self-esteem, physical literacy, physical activity, mediation, relationship

## Abstract

**Background:**

In light of the substantial decline in physical activity during college years, this study aims to examine the relationship between positive self-esteem, physical literacy, and physical activity in order to investigate the mechanisms for improving physical activity in college students and to provide a foundation for future interventions.

**Methods:**

A cross-sectional study design was employed in this study. A total of 5,184 Participants, aged between 17 and 21 years (*M* = 18.97, *SD* = 1.10), completed the Positive Version of Rosenberg Self-esteem Scales, Perceived Physical Literacy Instruments, and the International Physical Activity Questionnaires. A mediation model was utilized to explore the associations among the three concepts.

**Results:**

The three regression models were as follows: Physical literacy = 18.03 + 0.98 *Self-esteem, Physical activity = 43.23 + 0.16 *Self-esteem, and Physical activity = 28.18 + 0.11 *Physical literacy. Positive self-esteem, physical literacy, and physical activity were significantly linked with each other. Physical literacy mediated 26.93% of the effect, indicating a partial mediator in the relationship between positive self-esteem and physical activity.

**Conclusion:**

The mediating effect of physical literacy on the relationship between positive self-esteem and physical activity was identified. Our findings support the development of positive self-esteem and physical literacy in college physical education curricula as part of an overall program to address students’ physical inactivity at school and in the future. This study provides a new intervention perspective for improving physical inactivity in college students.

## 1. Introduction

Insufficient physical activity has long been recognized as a major risk factor for numerous preventable chronic diseases and mortality (Guthold et al., 2018). As physical activity levels continue to decline globally, insufficient physical activity now ranks as the fourth leading risk factor for death worldwide (World Health Organization, 2010). Acknowledging the numerous health benefits of physical activity, such as reduced risk of hypertension, breast cancer, colon cancer, diabetes, and cardiovascular disease, as well as its positive impact on mental health (World Health Organization, 2011; Sallis et al., 2016; ISPAH International Society for Physical Activity and Health, 2017; World Health Organization, 2022), the World Health Organization member states have committed to reducing the prevalence of insufficient physical activity by 10% by 2025 as one of the major global targets for non-communicable disease prevention and treatment (World Health Organization, 2013). Nevertheless, a study encompassing 1.9 million indicidualsacross 168 countries revealed minimal improvement in global physical activity levels to date (Guthold et al., 2018). Should this trend persist, the 2025 goal for increasing physical activity will prove challenging to attain. Hence, research and policies aimed at enhancing population-wide physical activity must be prioritized, rapidly implemented, and expanded. College students, in the final stage of their educationshould be encouraged to view physical activity as a vital component of their health (Whitehead, 2010; Whitehead, 2013). Given that college students typically spend their days on campus, universities play a cruical role in fostering and sustaining ongoing physical activity engagement (Ma et al., 2020b). Consequently, this study investigates the mechanisms that promote physical activity among college students and seeks to explore potential solutions to the escalating global issue of insufficient physical activity.

In revent years, physical literacy has emerged as a potential solution to the global decline in physical activity (Jurbala, 2015). This concept is defined as the motivation, confidence, physical competence, knowledge, and understanding to value and take responsibility for engagement in physical activities for life (Whitehead, 2010; Whitehead, 2019). Rooted in the philosophical monism of mind and body, physical literacy provides a framework for pursuing the harmonious unity of mental, physical, and environmental states (Whitehead, 2010; Whitehead, 2019). Three core dimensions— 1) motivation, 2) confidence and physical competence, and 3) interaction with the environment—comprise physical literacy (Whitehead, 2010). These mutually reinforcingattributes facilitate rewarding experiences in physical activity, leading to positive feedback and promoting overall physical literacy (Whitehead, 2010; Sum et al., 2016; Durden-Myers et al., 2018). Consequently, physical literacy has been acknwledged as the cornerstone for developing fundamental movement skills and establishing a foundation of lifetime physical activity participation (Keegan et al., 2013; Spengler and Cohen, 2015; Cairney et al., 2019; Caldwell et al., 2020b). A positive association exists between physical literacy and physical activity (Kwan et al., 2020; Ma et al., 2020b). Furthermore, physical literacy exerts beneficial effects on physical fitness (Caldwell et al., 2020a). While most of research has focused on the positive relevance of physical literacy to the physical domain, a few studies have also examined its connection to the mental domain. As a mind–body congruent concept, physical literacy correlates positively with mental health, well-being, and psychological resilience (Jefferies et al., 2019; Ma et al., 2021). This suggests that physical literacy may serve as aunique medium forconnecting body and mind at a level that encourages active physical activity.

Self-esteem, central to human mental health and well-being (Baumeister et al., 2003), is definded by Rosenberg as an indicator of self-acceptance, self-respect, and self-satisfaction (Winch and Rosenberg, 1965). Although a clear and consensual definition is elusive due to various conceptualization, most research agree that self-esteem emerges from one’s sense of self-concept (Shavelson et al., 1976; Greenwald et al., 2002). Within associative knowledge structure, self-concept arises from cognitive correlations between the self and one or more attributes, while self-esteem emerges from linking the self to affective evaluations of these correlations (Greenwald et al., 2002). Self-esteem has been characterized as a multidimensional hierarchical construct, with its multifaceted nature widely demonstrated (Shavelson et al., 1976; Marsh et al., 2006; Brunner et al., 2010; Rentzsch et al., 2016). This versatility enables the assessment of global self-esteem through global measures and specific self-esteem to predict specific behavioral outcomes (Swann et al., 2007). For instance, global self-esteem is a strong predictor of overall health (Rosenberg et al., 1995), including mental health, antidepressant medication usage (von Soest et al., 2016), and depressive symptoms (Orth et al., 2014). High or lowself-esteem significantly impacts an individual’s daily lives, with high self-esteem fostering greater perseverance and resilience when facing difficulties (Di Paula and Campbell, 2002). A meta-study identified cross-lagged effects, suggesting that low self-esteem significantly predicted depression and anxiety, which in turn implied significantly lower levels of physical activity (Sowislo and Orth, 2013). Given the positive correlation between physical activity and self-esteem, numerous studies have begun utilizing physical activity to enhance the psychological well-being of individuals with low self-esteem (Dale et al., 2019; Kucukibis and Gul, 2019). However, the mechanisms underlying these positive outcomes remain unidentified, warranting a more comprehensive investigation into whether high self-esteem can also promote physical activity.

**Figure 1:**
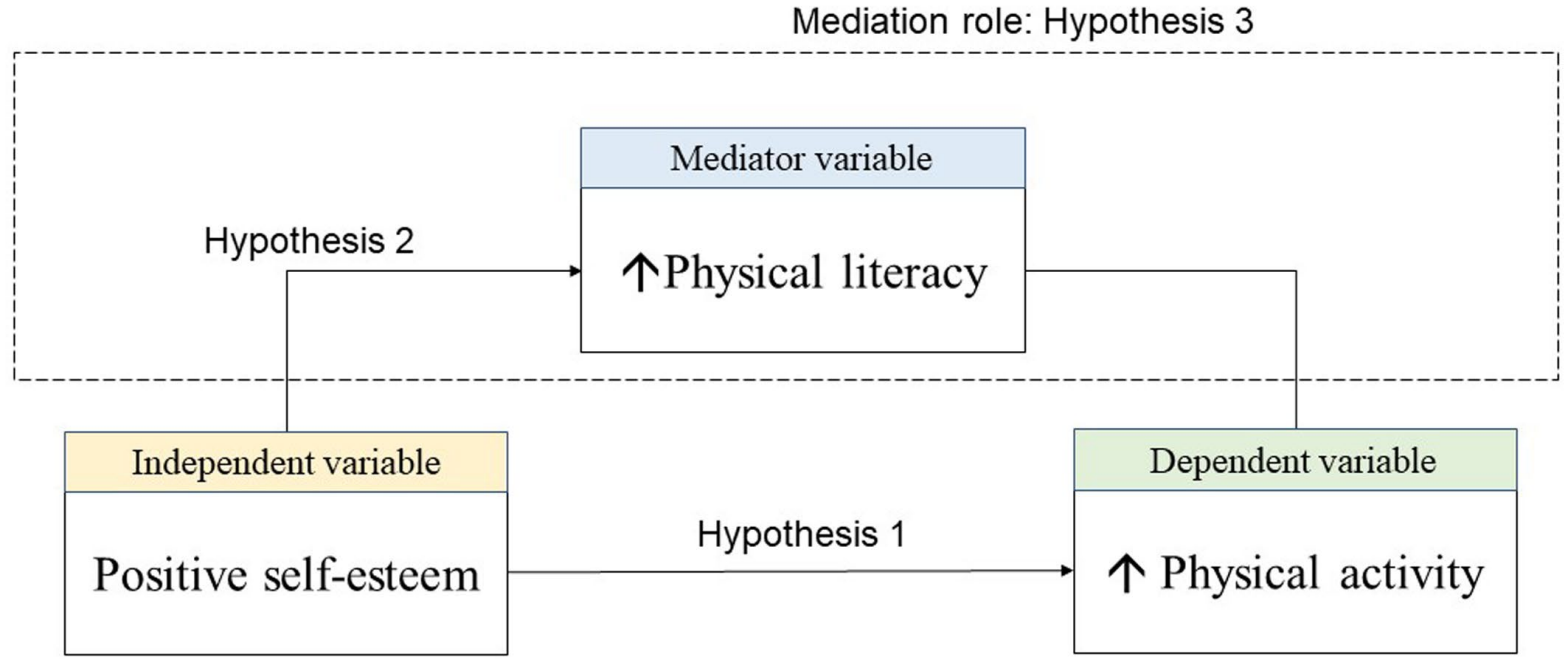
The mediation model.

Positive self-esteem, an alternative expression of overall self-esteem, is positively associated with learning motivation (Supple et al., 2013). This corresponds to one of the core concepts in physical literacy, which can be defined as a disposition characterized by a strong desire to employ motivation to improve one’s quality of life (Whitehead, 2010). As the link between body and mind, physical literacy is theoretically crucial for promoting physical activity (Ma et al., 2021). Positive self-esteem, with itsability to influence persistence under challenging circumstances, should also actively contribute to an individual’s attainment of physical and mental health (Niveau et al., 2021). These factors imply a correlation between positive self-esteem, physical literacy, and physical activity. Thus, a conceptual framework was designed to visualize the possible relationship between positive self-esteem, physical literacy and physical activity (see Figure 1). College students, in the final stage of their educational journey, must navigate the pressures of a changing environment and adapt to new patterns of socialization and learning. These changes may increase the incidence of physical and mental illness among college students (Regehr et al., 2013). As a result, physical activity levels among college students may decline rapidly. Given that exercise habits developed during this period can persist throughout life, intervention studies targeting this critical phase aim to employ cognitive and behavioral concepts to reduce psychological stress and subsequently increase physical activity in college students (Regehr et al., 2013). Self-esteem, encompassing self-concept, may play a pivotal role in this process (Niveau et al., 2021). Previous research has concentrated on utilizing physical activity to improve self-esteem and mental health. Based on the intrinsic nature of positive self-esteem, it is hypothesized to also influence the enhancement of physical activity (Liu et al., 2015; Rodrigues et al., 2022). To provide a theoretical basis for future intervention designspromoting physical activity among college students, this study aims to explore and clarify the contribution of positive self-esteem in facilitating physical activity and the role of physical literacy in this context. The hypotheses of this study are thus as follows (see Figure 1):

*Hypothesis 1*: Positive self-esteem positively influences physical activity.

*Hypothesis 2*: Positive self-esteem positively influences physical literacy.

*Hypothesis 3*: Physical literacy mediates the relationship between positive self-esteem and physical activity.

## 2. Method

### 2.1. Study design

This cross-sectional study was conducted at Jinan University, China using a convenience sampling. Questionnaires were distributed online,and all participants were informed of the details of the study and could withdraw from participation at any time, either temporarily or permanently. Ethical approval was obtained from the Jinan University IRB (JNUKY-2021-008).

### 2.2. Participants

A total of 5,835 undergraduate students participated in the study, with 5,184 completing the questionnaire, resulting in a response rate of 88.84%. Participants were first and second-year university students. The gender distribution was approximately equal [Male = 2,444 (47.15%); Female = 2,740 (52.85%)]. The students’ ages ranged from 17 to 21 years (Total_age_: *M* = 18.97, *SD* = 1.10; Male_age_: *M* = 19.51, *SD* = 0.89; Female_age_: *M* = 18.67, *SD* = 1.09), with most being 19 years old (38.01%). Nearly half the participants were first-year students (*N* = 2,662, 50.58%), while the rest were second-year students (*N* = 2,522, 49.42%). The participants’ study streams were primarily liberal arts (*N* = 1,831, 35.32%) and science (*N* = 2,676, 51.62%), followed by medicine (*N* = 480, 9.26%), and law (*N* = 197, 3.80%).

### 2.3. Measures

The Rosenberg Self-esteem Scale was used to measure participants’ self-esteem level (Rosenberg, 1965). With the accumulation of research data and the use of analytical techniques, exploratory and validation analyses revealed that overall self-esteem was divided into two dimensions: positive self-esteem and negative self-esteem (Schmitt and Allik, 2005; Boduszek et al., 2013). These two dimensions resulted from method effects (Urbán et al., 2014). To focus more on the self-esteem scale itself rather than wording distinctions, a later study created a 10 items unidimensional positive self-esteem scale by transposing the negative expressions to positive statements (eg., “I feel that I have a number of good qualities” and “I feel I do have much to be proud of”) (Greenberger et al., 2003). This new version of the positive self-esteem scale was used in this study. The scale had been translated to the Chinese and demonstrated good psychometric properties in Chinese university students with a Cronbach’s alpha (*α*) of 0.87 (Chen et al., 2015).

Physical activity levels of the students were assessed using the Chinese Version of International Physical Activity Questionnaire (short version), which included the number of hours and frequency of moderate and vigorous intensity physical activity (MVPA) during the week (Qu and Li, 2004). Data were collected in minutes per week for each physical activity intensity. Minutes were then converted to metabolic equivalent (MET) values using the MET-minutes computation formulation (Booth, 2000; Craig et al., 2003). The Chinese version of the questionnaire was assessed using test–retest method (*p* < 0.05) and the correlation test established by the Caltrac accelerometer (*p* < 0.05, *r* = 0.50), demonstrating good validity and reliability for college students (Qu and Li, 2004).

Physical literacy was assessed using the Simplified Chinese Version of Perceived Physical Literacy Instrument (Ma et al., 2020a), which included three dimensions: 1) motivation (eg., “I appreciate myself or others doing sports”), 2) confidence and physical competence (eg., “I am able to apply learnt motor skills to other physical activities”), and 3) interaction with the environment (eg., “I have strong communication skills”). The instrument was developed based on Whitehead’s definition of physical literacy and measured college students’ attitudes toward physical activity and the extent to which they took responsibility for their bodies (Whitehead, 2010). The Simplified Chinese Version of Perceived Physical Literacy Instrument was shown to be reliable and valid for measuring physical literacy among Chinese university students, with a Cronbach’s alpha (α) of 0.86 and confirmatory factor analysis (factor loadings ranged from 0.60 to 0.92) (Ma et al., 2020a,b).

### 2.4. .Statistical analysis

Data were analyzed using SPSS 25 and PROCESS macro 3.5 (Model 4) (Rockwood and Hayes, 2017). The reliability of all measurement instruments in this study was calculated using Cronbach’s alpha. Prior to analysis, normality, homoscedasticity, and linearity were examined and found to be supported. The correlation among each variable was derived from the bivariate Pearson’s product–moment correlation coefficient (*r*). Standard regression and the bootstrap method were used to test the mediational hypothesis. Compared to traditional stepwise testing (Baron and Kenny, 1986) and the Sobel method (Sobel, 1982), bootstrap is less demanding on the sample and more sensitive in determining the model. In this study, 5,000 bootstrap samples were used. Positive self-esteem served as the predictor, physical literacy as the mediator, and physical activity as the outcome variable. Age, gender, and grade were set as covariates based on previous studies (Zuckerman, 1989; Bremer et al., 2020; Ma et al., 2020b; Schlund et al., 2021). Direct and indirect effects were calculated to determine the mediation model results. Confidence intervals (CI) that did not contain zero were considered to be significant. Statistical significance was set at *p* < 0.05.

## 3. Results

### 3.1. Linear regression for testing hypotheses 1 and 2

**Figure 2:**
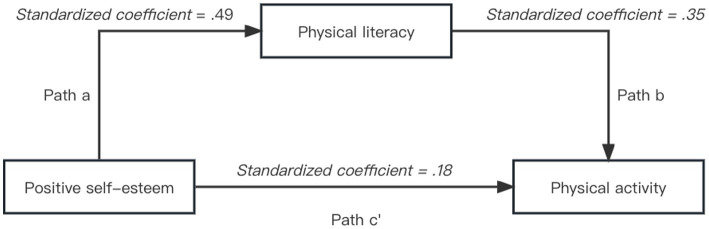
Graphical illustration of the mediation model of physical literacy for positive self-esteem and physical activity.

Standard linear regression was employed to investigate the relationships among positive self-esteem, physical activity, and physical literacy (Table 1). All correlations were positive and significant, indicating that the these three variables were significantly related to each other. The three regression models were as follows: Physical literacy = 18.03 + 0.98 * Self-esteem, Physical activity = 43.23 + 0.16 * Self-esteem, and Physical activity = 28.18 + 0.11 * Physical literacy. Figure 2 presents a graphical illustration of the simple mediation model with unstandardized coefficients (*β*) and standard error (*SE*).

**Table 1:** Results of the standard linear regression analysis among positive self-esteem, physical literacy, and physical activity.

	*β*	*F*	95% CI	*R*	Δ*R*^2^
Positive self-esteem					
Physical literacy	18.03^a^	179.26	13.63, 22.43	0.48	0.24
Physical activity	43.24^a^	92.98	42.08, 44.40	0.33	0.17
Physical literacy					
Physical activity	28.18^a^	750.07	27.98, 28.39	0.36	0.13

a Correlation is significant at the 0.01 level (two tailed).

### 3.2. Mediation analysis for testing hypotheses 3

The bootstrap method was used to examine the mediating effect of physical literacy on the relationship between positive self-esteem and physical activity, with age, gender and grade as covariates. The mediation model displayed a non-zero boot CI (53.05, 64.06) with a total effect of 58.40 and an indirect effect of 15.74. This result indicated that physical literacy mediated 26.93% of the effect, suggesting a partial mediator in the relationship between positive self-esteem and physical activity.

## 4. Discussion

The study explored the relationship between positive self-esteem and physical activity and examined the mediating effect of physical literacy on this relationship among college students. The results indicated significant associations among these three concepts. The mediating effect of physical literacy contributes to understanding the association between positive self-esteem and physical activity in this population.

Physical activity interventions have found to effectively increase participants’ self-esteem (Dale et al., 2019; Kucukibis and Gul, 2019). In accordance with the definition of self-esteem and the pathway from positive self-esteem to physical activity, this study further demonstrated that positive self-esteem itself was positively correlated with physical activity and served as an independent variable to effectively predict physical activity. Specifically, individuals with good exercise habits and healthy physical activity levels tend to have high self-esteem and self-concept. Likewise, high self-esteem would, in turn, increased physical activity. This finding is consistent with previous studies that self-esteem is a multidimensional concept with relevance across various domains (Niveau et al., 2021). This connection supports the diea that different dimensions of global self-esteem can be subdivided into specific directions for different predictive tasks under various domains, such as positive self-esteem for actively engaging in physical activity.

Although a significant positive correlation was found between positive self-esteem and physical activity, the correlation coefficient remained low. One potential mechanism linking them was through physical literacy. As a multidimensional concept, physical literacy represents the integration of body and mind (Whitehead, 2010; Whitehead, 2019; Ma et al., 2021), aligning with the link between positive self-esteem and physical activity at the mental and physical levels. Our results supported the idea that a significant positive correlation existed between positive self-esteem and physical literacy. According to Whitehead’s model, physical literacy develops from three domains: affective, physical, and cognitive. At the macro level, physical literacy emphasizes the indivisibility of body and mind, with several dimensions interacting with each other. At the micro level, physical literacy emphasizes lifelong movement and positive attitudes (Whitehead, 2019). Meanwhile, self-esteem is rooted in self-acceptance, self-respect, and individual’s evaluation of their self-concept in specific domains shaped by experiences in different environments (Rosenberg, 1965; Shavelson et al., 1976; Greenwald et al., 2002). Commonalities at the level of cognition, interaction with the environment, and positive attitudes toward life may underlie the association between positive self-esteem and physical literacy.

Physical literacy had been shown to be inextricably linked to physical activity (Ma et al., 2020b). Additionally, the model in this study supports the hypothesis that physical literacy, as a mediator factors, significantly influences the connection betweenn mind and body. Therefore, when promoting physical activity, positive self-esteem must pass through the pathway of physical literacy. As a promising new mechanism, the mediational model of physical literacy may partially explain the relationship between positive self-esteem and physical activity. Th connection between these positive self-esteem, physical literacy, and physical activity in the mediation model can provide a new approach for improving physical activity in physical education. For instance, incorporating elements of positive self-esteem and core dimensions of physical literacy, such as motivation, confidence and physical competence., in physical education not only helps students improve their physical health, but also enhances their mental health on a spiritual level (Whitehead, 2010; Supple et al., 2013; Choi et al., 2018; Ma et al., 2021). This aligns with the concept of physical literacy, at a time when it is widely brought to the interdisciplinary field of exploration, physical literacy should be considered intrinsic to human flourishing (Durden-Myers et al., 2018). Given that physical and mental health issues are particularly prominent among college students, an integrative concept must be utilized to address the problem. Our findings also support the idea that physical literacy may be an optimal way to not only alleviate psychological problems, but also to improve students’ physical activity level to achieve physical and mental health.

Physical inactivity is a leading global problem (Guthold et al., 2018). College students are about to enter society, and their college years represent the final opportunity to increase their physical activity levels and develop lifelong exercise habits during their educational years (Whitehead, 2013). In the long run, physical health problems caused by physical inactivity can affect students’ future productivity and overall well-being (Beauchamp et al., 2018). Insufficient physical activity also significantly impacts students’ academic performance and efficiency (Ma et al., 2020b). In this context, the present study provides a new perspective for addressing this issue by encouraging the development of physical literacy and promoting physical activity through increased positive self-esteem. This study has practical implications for developing physical education programs at universities. Building on studies supporting the benefits of introducing physical literacy into college curricula (Whitehead, 2019; Ma et al., 2021), this study suggests college physical education programs that increase positive self-esteem and physical literacy to improve physical inactivity among college students, including syllabus elements that are encouraging and foster positive self-esteem.

## 5. Strength

This study represents the first investigation of the mediating factor between positive self-esteem and physical activity. The results remained significant after controlling for age, gender and grade, suggesting that enhancing positive self-esteem as a foundation, alongside improving physical literacy, can effectively increasing college students’ physical activity levels. This mediation pathway offers robust support for the design of future interventions.

## 6. Limitation

Firstly, the participants in this study were Chinese university students. Although physical inactivity among college students is a global issue, differences in education systems across countries result in distinct physical education programs, which may lead to variability in mediated relationships among different populations. Consequently, the findings of this study have limited applicability for guiding countries with diverse education systems. Secondly, the study was conducted at a single university and using convenience sampling, with indicators extracted from subjective questionnaires, which affects the generalizability of the results. Thirdly, while the study’s findings support the hypothesized relationships described in the existing literature, causal statements regarding the association among positive self-esteem, physical literacy, and physical activity should be made with caution. The cross-sectional findings necessitate further experimental research to validate the observed causal inferences.

## 7. Conclusion

This study explores the relationship among positive self-esteem, physical literacy, and physical activity, constructing a mediated model through the path between them, suggesting a potential solution for insufficient physical activity. The relationship among the three variables are positively correlated, with physical literacy serving as a mediating factor. As an increasing number of college students experiencing physical inactivity due to a lack of lifelong exercise habits, many universities are intensifying their focus on physical education classes. Our findings support the integration of positive self-esteem and physical literacy into college physical education curricula as part of a comprehensive program to address students’ physical inactivity during their education and into the future. This study offers a novel intervention perspective for ameliorating physical inactivity in college students.

## Data availability statement

The raw data supporting the conclusions of this article will be made available by the authors, without undue reservation.

## Ethics statement

The studies involving human participants were reviewed and approved by Jinan University IRB (JNUKY-2021-008). The patients/participants provided their written informed consent to participate in this study.

## Author contributions

XS and T-YG: conceptualization. R-SM: methodology, formal analysis, and writing—original draft preparation. R-SM and XS: validation. R-SM, T-YG, DT, HZ, and H-LD: writing—review and editing. All authors contributed to the article and approved the submitted version.

## Funding

This research is supported by the Guangdong Office of Philosophy and Social Science (GD21YTY04) and partially supported by the Fundamental Research Funds for the Central Universities (21JNQN14).

## Conflict of interest

The authors declare that the research was conducted in the absence of any commercial or financial relationships that could be construed as a potential conflict of interest.

## Publisher’s note

All claims expressed in this article are solely those of the authors and do not necessarily represent those of their affiliated organizations, or those of the publisher, the editors and the reviewers. Any product that may be evaluated in this article, or claim that may be made by its manufacturer, is not guaranteed or endorsed by the publisher.

## References

[ref1] Baron R. M. Kenny D. A. (1986). The moderator-mediator variable distinction in social psychological research. Conceptual, strategic, and statistical considerations. J. Pers. Soc. Psychol. 51, 1173–1182. doi: 10.1037/0022-3514.51.6.1173, PMID: 3806354

[ref2] Baumeister R. F. Campbell J. D. Krueger J. I. Vohs K. D. (2003). Does high self-esteem cause better performance, interpersonal success, happiness, or healthier lifestyles? Psychol. Sci. Public Interes. 4, 1–44. doi: 10.1111/1529-1006.01431 26151640

[ref3] Beauchamp M. R. Puterman E. Lubans D. R. (2018). Physical inactivity and mental health in late adolescence. JAMA Psychiat. 75, 543–544. doi: 10.1001/jamapsychiatry.2018.0385, PMID: 29710114

[ref4] Boduszek D. Hyland P. Dhingra K. Mallett J. (2013). The factor structure and composite reliability of the Rosenberg self-esteem scale among ex-prisoners. Pers. Individ. Dif. 55, 877–881. doi: 10.1016/j.paid.2013.07.014

[ref5] Booth M. (2000). Assessment of physical activity: an international perspective. Res. Q. Exerc. Sport 71, 114–120. doi: 10.1080/02701367.2000.11082794 25680021

[ref6] Bremer E. Graham J. D. Cairney J. (2020). Outcomes and feasibility of a 12-week physical literacy intervention for children in an afterschool program. Int. J. Environ. Res. Public Health 17:3129. doi: 10.3390/ijerph17093129, PMID: 32365870 PMC7246927

[ref7] Brunner M. Keller U. Dierendonck C. Reichert M. Ugen S. Fischbach A.. (2010). The structure of academic self-concepts revisited: the nested Marsh/Shavelson model. J. Educ. Psychol. 102, 964–981. doi: 10.1037/a0019644

[ref8] Cairney J. Kiez T. Roetert E. P. Kriellaars D. (2019). A 20th-century narrative on the origins of the physical literacy construct. J. Teach. Phys. Educ. 38, 79–83. doi: 10.1123/jtpe.2018-0072

[ref9] Caldwell H. A. T. Di Cristofaro N. A. Cairney J. Bray S. R. Macdonald M. J. Timmons B. W. (2020a). Physical literacy, physical activity, and health indicators in school-age children. Int. J. Environ. Res. Public Health 17, 1–12. doi: 10.3390/ijerph17155367, PMID: 32722472 PMC7432049

[ref10] Caldwell H. A. T. Wilson A. Mitchell D. Timmons B. W. (2020b). Development of the physical literacy environmental assessment (PLEA) tool. PLoS One 15, e0230447–e0230415. doi: 10.1371/journal.pone.0230447, PMID: 32182272 PMC7077881

[ref11] Chen F. Bi C. Mengfei H. (2015). The reliability and validity of the Chinese version of the revised-positive version of Rosenberg self-esteem scale. Adv. Psychol. 5:531535, 531–535. doi: 10.12677/AP.2015.59068

[ref12] Choi S. M. Sum R. K. W. Leung E. F. L. Ng R. S. K. (2018). Relationship between perceived physical literacy and physical activity levels among Hong Kong adolescents. PLoS One 13, e0203105–e0203111. doi: 10.1371/journal.pone.0203105, PMID: 30148876 PMC6110504

[ref13] Craig C. L. Marshall A. L. Sjöström M. Bauman A. E. Booth M. L. Ainsworth B. E.. (2003). International physical activity questionnaire: 12-country reliability and validity. Med. Sci. Sports Exerc. 35, 1381–1395. doi: 10.1249/01.MSS.0000078924.61453.FB, PMID: 12900694

[ref14] Dale L. P. Vanderloo L. Moore S. Faulkner G. (2019). Physical activity and depression, anxiety, and self-esteem in children and youth: an umbrella systematic review. Ment. Health Phys. Act. 16, 66–79. doi: 10.1016/j.mhpa.2018.12.001

[ref15] Di Paula A. Campbell J. D. (2002). Self-esteem and persistence in the face of failure. J. Pers. Soc. Psychol. 83, 711–724. doi: 10.1037/0022-3514.83.3.711, PMID: 12219864

[ref16] Durden-Myers E. J. Whitehead M. E. Pot N. (2018). Physical literacy and human flourishing. J. Teach. Phys. Educ. 37, 308–311. doi: 10.1123/jtpe.2018-0132

[ref17] Greenberger E. Chen C. Dmitrieva J. Farruggia S. P. (2003). Item-wording and the dimensionality of the Rosenberg self-esteem scale: do they matter? Pers. Individ. Dif. 35, 1241–1254. doi: 10.1016/S0191-8869(02)00331-8

[ref18] Greenwald A. G. Banaji M. R. Rudman L. A. Farnham S. D. Nosek B. A. Mellott D. S. (2002). A unified theory of implicit attitudes, stereotypes, self-esteem, and self-concept. Psychol. Rev. 109, 3–25. doi: 10.1037/0033-295X.109.1.3, PMID: 11863040

[ref19] Guthold R. Stevens Gretchen A. Riley L. M. Bull F. C. (2018). Worldwide trends in insufficient physical activity from 2001 to 2016: a pooled analysis of 358 population-based surveys with 1·9 million participants. Lancet Glob. Heal. 6, e1077–e1086. doi: 10.1016/S2214-109X(18)30357-7, PMID: 30193830

[ref20] ISPAH International Society for Physical Activity and Health (2017). The Bangkok declaration on physical activity for Global Health and sustainable development. Br. J. Sports Med. 51, 1389–1391. doi: 10.1136/bjsports-2017-098063, PMID: 28642224

[ref21] Jefferies P. Ungar M. Aubertin P. Kriellaars D. (2019). Physical literacy and resilience in children and youth. Front. Public Heal. 7:346. doi: 10.3389/fpubh.2019.00346, PMID: 31803709 PMC6877541

[ref22] Jurbala P. (2015). What is physical literacy, really? Quest 67, 367–383. doi: 10.1080/00336297.2015.1084341

[ref23] Keegan R. Keegan S. Daley S. Ordway C. Edwards A. Getting Australia Moving: Establishing a Physically Literate Active Nation (Game Plan). University of Canberra; Canberra, (2013).

[ref24] Kucukibis H. F. Gul M. (2019). The relationship between attitudes towards physical activity and self-esteem of high school students. Asian J. Educ. Train. 5, 70–73. doi: 10.20448/journal.522.2019.51.70.73

[ref25] Kwan M. Y. W. Graham J. D. Healey C. Paolucci N. Brown D. M. (2020). Stopping the drop: examining the impact of a pilot physical literacy-based intervention program on physical activity behaviours and fitness during the transition into university. Int. J. Environ. Res. Public Health 17, 1–12. doi: 10.3390/ijerph17165832, PMID: 32806584 PMC7459702

[ref26] Liu M. Wu L. Ming Q. (2015). How does physical activity intervention improve self-esteem and self-concept in children and adolescents? Evidence from a Meta-analysis. Wallander JL, ed. PLoS One 10:e0134804. doi: 10.1371/journal.pone.0134804 26241879 PMC4524727

[ref27] Ma R. Liu T. Raymond Sum K. W. Gao T. Li M. Choi S. M.. (2021). Relationship among physical literacy, mental health, and resilience in college students. Front. Psychiatry 12:12. doi: 10.3389/fpsyt.2021.767804, PMID: 34966305 PMC8710533

[ref28] Ma R. S. Sum R. K. W. Hu Y. N. Gao T. Y. (2020a). Assessing factor structure of the simplified Chinese version of perceived physical literacy instrument for undergraduates in mainland China. J. Exerc. Sci. Fit. 18, 68–73. doi: 10.1016/j.jesf.2020.01.001, PMID: 31998384 PMC6965736

[ref29] Ma R. S. Sum R. K. W. Li M. H. Huang Y. Niu X. L. (2020b). Association between physical literacy and physical activity: a multilevel analysis study among chinese undergraduates. Int. J. Environ. Res. Public Health 17, 1–12. doi: 10.3390/ijerph17217874, PMID: 33121068 PMC7663683

[ref30] Marsh H. W. Trautwein U. Ludtke O. Koller O. Baumert J. (2006). Integration of multidimensional self-concept and Core personality constructs: construct validation and relations to well-being and achievement. J. Pers. 74, 403–456. doi: 10.1111/j.1467-6494.2005.00380.x, PMID: 16529582

[ref31] Niveau N. New B. Beaudoin M. (2021). Self-esteem interventions in adults – a systematic review and Meta-analysis. J. Res. Pers. 94:104131. doi: 10.1016/j.jrp.2021.104131

[ref32] Orth U. Robins R. W. Widaman K. F. Conger R. D. (2014). Is low self-esteem a risk factor for depression? Findings from a longitudinal study of Mexican-origin youth. Dev. Psychol. 50, 622–633. doi: 10.1037/a0033817, PMID: 23895172 PMC3815504

[ref33] Qu N. Li K. (2004). Study on the reliability and validity of international physical activity questionnaire (Chinese vision, IPAQ). Zhonghua Liu Xing Bing Xue Za Zhi 25, 265–268. Available at: http://europepmc.org/abstract/MED/15200945. PMID: 15200945

[ref34] Regehr C. Glancy D. Pitts A. (2013). Interventions to reduce stress in university students: a review and meta-analysis. J. Affect. Disord. 148, 1–11. doi: 10.1016/j.jad.2012.11.026, PMID: 23246209

[ref35] Rentzsch K. Wenzler M. P. Schütz A. (2016). The structure of multidimensional self-esteem across age and gender. Pers. Individ. Dif. 88, 139–147. doi: 10.1016/j.paid.2015.09.012

[ref36] Rockwood N. J. Hayes A. F. (2017). MLmed: An SPSS Macro for Multilevel Mediation and Conditional Process Analysis, vol. 19 Available at: www.afhayes.com.

[ref37] Rodrigues F. Faustino T. Santos A. Teixeira E. Cid L. Monteiro D. (2022). How does exercising make you feel? The associations between positive and negative affect, life satisfaction, self-esteem, and vitality. Int. J. Sport Exerc. Psychol. 20, 813–827. doi: 10.1080/1612197X.2021.1907766

[ref38] Rosenberg M. Rosenberg Self-Esteem Scale. Princeton, NJ: Princeton University Press (1965).

[ref39] Rosenberg M. Schooler C. Schoenbach C. Rosenberg F. (1995). Global self-esteem and specific self-esteem: different concepts, different outcomes. Am. Sociol. Rev. 60:141. doi: 10.2307/2096350

[ref40] Sallis J. F. Bull F. Guthold R. Heath G. W. Inoue S. Kelly P.. (2016). Progress in physical activity over the Olympic quadrennium. Lancet 388, 1325–1336. doi: 10.1016/S0140-6736(16)30581-5, PMID: 27475270

[ref41] Schlund A. Reimers A. K. Bucksch J. Brindley C. Schulze C. Puil L.. (2021). Do intervention studies to promote physical activity and reduce sedentary behavior in children and adolescents take sex/gender into account? A systematic review. J. Phys. Act. Health 18, 461–468. doi: 10.1123/jpah.2020-0666, PMID: 33668018

[ref42] Schmitt D. P. Allik J. (2005). Simultaneous administration of the Rosenberg self-esteem scale in 53 nations: exploring the universal and culture-specific features of global self-esteem. J. Pers. Soc. Psychol. 89, 623–642. doi: 10.1037/0022-3514.89.4.623, PMID: 16287423

[ref43] Shavelson R. J. Hubner J. J. Stanton G. C. (1976). Self-concept: validation of construct interpretations. Rev. Educ. Res. 46, 407–441. doi: 10.3102/00346543046003407

[ref44] Sobel M. E. (1982). An interactive calculation tool for mediation tests, calculation for the Sobel test, asymptotic intervals for indirect effects in structural equations models. Sociol Method. 13, 290–312. doi: 10.2307/270723

[ref45] Sowislo J. F. Orth U. (2013). Does low self-esteem predict depression and anxiety? A meta-analysis of longitudinal studies. Psychol. Bull. 139, 213–240. doi: 10.1037/a0028931 22730921

[ref46] Spengler J. O. Cohen J. Physical literacy: A global environmental scan. Washington, DC: Aspen Institute Sports & Society Program. (2015).

[ref47] Sum R. K. W. Ha A. S. C. Cheng C. F. Chung P. K. Yiu K. T. Kuo C. C.. (2016). Construction and validation of a perceived physical literacy instrument for physical education teachers. PLoS One 11, 1–10. doi: 10.1371/journal.pone.0155610, PMID: 27195664 PMC4873233

[ref48] Supple A. J. Su J. Plunkett S. W. Peterson G. W. Bush K. R. (2013). Factor structure of the Rosenberg self-esteem scale. J. Cross-Cult. Psychol. 44, 748–764. doi: 10.1177/0022022112468942

[ref49] Swann W. B. Chang-Schneider C. Larsen M. C. K. (2007). Do people’s self-views matter? Self-concept and self-esteem in everyday life. Am. Psychol. 62, 84–94. doi: 10.1037/0003-066X.62.2.84, PMID: 17324034

[ref50] Urbán R. Szigeti R. Kökönyei G. Demetrovics Z. (2014). Global self-esteem and method effects: competing factor structures, longitudinal invariance, and response styles in adolescents. Behav. Res. Methods 46, 488–498. doi: 10.3758/s13428-013-0391-5, PMID: 24061931 PMC3947716

[ref51] von Soest T. Wichstrøm L. Kvalem I. L. (2016). The development of global and domain-specific self-esteem from age 13 to 31. J. Pers. Soc. Psychol. 110, 592–608. doi: 10.1037/pspp0000060, PMID: 26167796

[ref52] Whitehead M. Physical Literacy: Throughout the Lifecourse. Routledge, England; (2010)

[ref53] Whitehead M. Definition of physical literacy and clarification of related issues. ICSSPE Bulletin (2013); 65. Frankfurt

[ref54] Whitehead M. Physical literacy across the world. Routledge England; (2019).

[ref55] Winch R. F. Rosenberg M. (1965). Society and the adolescent self-image. Soc. Forces 44:255. doi: 10.2307/2575639

[ref56] World Health Organization. Global Recommendations on Physical Activity for Health; (2010). World Health Organization, Geneva. 26180873

[ref57] World Health Organization. Global Recommendations on Physical Activity for Health: 18–64 years old; (2011). World Health Organization, Geneva.

[ref58] World Health Organization. Global Action Plan for the Prevention and Control of Noncommunicable Diseases 2013–2020 (2013): 102. World Health Organization, Geneva

[ref59] World Health Organization (2022). Physical Activity. Available at: https://www.who.int/news-room/fact-sheets/detail/physical-activity

[ref60] Zuckerman D. M. (1989). Stress, self-esteem, and mental health: how does gender make a difference? Sex Roles 20, 429–444. doi: 10.1007/BF00288001

